# The Relationship Between Religious Orientation, and Gender With a Cognitive Distortion

**Published:** 2014

**Authors:** Leili Amirsardari, Shafie Azari, Ahmad Esmali Kooraneh

**Affiliations:** 1 Department of Psychology, Science and Research Branch, Islamic Azad University, West Azarbayjan, Iran.; 2 Department Of Psychology, Payame Noor University, Tehran, Iran.; 3 Assistant Professor, Department of Humanistic Science, School of literature, Maragheh University, Maragheh, Iran

**Keywords:** Cognitive, Cognitive Distortions, Gender, Religious Orientation

## Abstract

**Objective:** The objective of this study was to determine the relationship between religious orientation (intrinsic–external) and cognitive distortions.

**Methods:** General design of this study considered as a descriptive and correlational method. Universal population in this research consist all students of the Urmia Azad University, which were studying during 2012 and 2013 (n = 250). All respondents filled the Alports religious and cognitive distortions questionnaires. The answers were analyzed with step by step regression and correlation method.

**Results: **The research showed a significant relationship between the religious orientation and cognitive distortions (p < 0.005) (p < 0.001).

**Conclusion:** The results suggest that religious orientation is an important factor in cognitive distortions and individuals with intrinsic religious orientation have less cognitive distortion.

## Introduction

The main factor in human distinguish is religion (1). So far there has been no sign of the animal’s religious life; we nocannot find any tribe without religion in human being (2). The need of human beings to a life with religion is back to several centuries ago, so that mankind in the beginning of his life has felt the need of powerful protection origin and felt dependence on this origin (3). Historically the oldest kind of human beings have had a religious pattern, and if divine system had not been created, the human would create it besides. If they had not a god to worship, they would make it so they pray for sculptures, satanic forces, sun and hallucination merely for reaching peacefulness (4).

Based on the psychological definition of religion by Carl Gustav Jung (1875-1961): “Undoubtedly, religion is the best demonstration of the human spirit” or based on Kerfman: “Religion has root in the human desire for elevation it’s dignity” or Samuel tells: “Religion is the statement of belief in mysterious forces caused by feelings such as fear and worship;” or Freud says, “it's the human exploration to find the heavenly soothing, which help him to conquer the stressful events of life.” From psychological viewpoint, religion is personal feature and has origin in inner side of a person (5). From the viewpoint of Allport, religious orientation contains intrinsic and extrinsic aspects. They believe that it is the most acceptable method in distinction of these both poles of religion, that we say a person with extrinsic religion apply it, while a person with intrinsic religion live with (6).

Individual with intrinsic religion who believes that religion is in their origin has more mental health than whom with extrinsic religious and has accepted that the religion is the way of achievement to other objects (7). Individuals with intrinsic religious orientation are more reasonable in terms of cognitive, and have more mental health and are more satisfied with life (6). Those who have extrinsic religion from divine philosophically, come toward God and are far from themselves (8). So recently, research on the religious orientation and mental health has been increased (6). 

Cognitive approach is one of the most influential psychological theories in the field of mental health disorders, therefore the core of this approach is cognition, and refers to this reality that the view of two individual from the same event is different that shows the difference in thought of these two individuals. In fact, the way we think, determines how we feel (9).

It is believed that the negative thoughts and cognitive distortions cause the anxious and depressed mood in the individuals with these disorders. According to the cognitive therapy, beliefs and thoughts are the cause of anxiety and concern, to be concerned of the incident first, it should be interpreted, and then consider the meaning. This point of view is important to have that changing mind can change your feelings (5).

The term of cognitive distortions is used to describe a special line of thinking that is distorted and had imaginary content (10). Ten cognitive distortions contain the followings:

1. All or nothing thinking,
2. Overgeneralization,
3. Mental filter,
4. Disqualifying the positive,
5. Jumping to conclusions,
6. Magnification (catastrophizing) or minimization,
7. emotional reasoning,
8. Should statements,
9. Labeling and mislabeling,
10. Personalization (11).

The aim of the research is studying the relationship between intrinsic and extrinsic religious orientation, cognitive distortion and gender.

## Materials and Methods

The method used in the study is descriptive one, and correlation statistical analyses also used in this study. The population of this study include students (n = 750). Samples were selected based on a cluster random sampling method (we use cluster sampling method), because large community does not make it possible to provide people randomly, so that individuals have an equal chance to be selected. Based on Morgan table; 254 undergraduate students were selected from three college of engineering, paramedical, and science in Islamic Azad University of Urmia in 2012-2013. From 254 individual, 250 cases were fully responds to the questionnaire. The sample included 250 persons (127 females and 123 males) (50.8% females and 49.2% males) and average of their age was 22.86 years old. The variable of marital status was not examined and all of them were Muslim.

***Instrument***

We used two questionnaires that include the Allport Ross religious orientation scale and cognitive distortions questionnaire. Cronbach alpha coefficient in the first subscale is 0.76 and its stability is 0.71, also in the second subscale, these values are 0.87 and 0.80, respectively (12). It scored through Likert method that also done based on another study. Validity of it was 0.71. This test in category method, measures the intrinsic and extrinsic religious orientation (13).

*Cognitive distortion*

The questionnaire contains 20 phrases, which assess the cognitive distortion of Albert Allis and each irrational thoughts have two specific phrases that scored through Likert scale, and its Cronbach’s alpha is 0.80 (14).

*Ethics*

Anonymous questionnaire was prepared to ensure that the results obtained by the participants in this study would not hurt to anyone.

Data includes descriptive statistics for distribution of data as mean and standard deviation, analytical statistics as correlation, multiple regression and its subscales among men and women, were analyzed using SPSS for Windows (version 18; SPSS Inc., Chicago, IL, USA). Statistical significance was evaluated with alpha levels of 0.05 and 0.01.

## Results

As shown in table 1, it is important to mention that among cognitive distortions, the highest average in relation to labeling, and for religious orientation, the greatest mean, relates to exterior religious orientation.

According to table 2, there is a positive relationship between extrinsic religious orientation with the thinking of all or nothing and disregarding the positive (p < 0.01) and also there is a negative and significant relationship between extrinsic religion orientation and must be personalize (p < 0.05).

According to second hypothesis, a significant positive relationship between extrinsic religious orientation thinking and disregard all and any positive in p < 0.01 there is also a significant negative relationship between extrinsic religious orientation and the words of should someone 0.05 level there. Thus, the first hypothesis is confirmed.

**Table 1:** Total descriptive determinant of the study has provided

**Variable**	**Mean**	**SD** †
**Age**	22.86	03.12
**All or nothing thinking**	04.57	03.45
**Overgeneralization**	03.53	03.48
**Mental filter**	02.70	02.87
**Discounting the positive**	02.68	02.74
**Jumping to conclusion**	06.95	01.99
**Emotional reasoning**	07.25	01.78
**Magnifying or minimizing**	07.09	01.89
**Should statements**	02.55	02.49
**Labeling**	07.61	02.04
**Personalization**	07.11	01.85
**Intrinsic orientation**	14.65	12.61
**Extrinsic orientation**	23.37	17.13

† Standard deviation

**Table 2:** Correlation between cognitive distortions and its subscales with religious orientation (intrinsic and external)

**Variable**	**Extrinsic orientation**	**Intrinsic orientation**
**Age**	0.09**	0.08**
**All or nothing**	0.27**	0.03**
**Overgeneralization**	0.01**	0.04**
**Mental filter**	0.28**	0.27**
**Discounting the positive**	0.07**	0.03**
**Jumping to conclusion**	0.01**	0.07**
**Emotional reasoning**	0.01**	0.06**
**Magnifying or minimizing**	0.01**	0.04**
**Should statements**	0.19**	0.27**
**Labeling**	0.08**	0.02**
**Personalization**	0.14**	0.01**

* P < 0.05,

** P < 0.01

Furthermore, there is a significant negative relationship between extrinsic religious orientation (p < 0.05). Therefore, the first hypothesis is confirmed. For the second theory (there is a significant relationship between intrinsic orientation and cognitive distortion) we used second table. According to table, there is a negative significant relationship between intrinsic orientation and mental filter and should expressed in p < 0.01, therefore the second theory is confirmed.

The third hypothesis expresses that there is no relationship between age and religious orientation. Therefore, the third theory is not confirmed. Regarding the table, the forth theory shows that a significant relationship between religious orientation and cognitive distortion is confirmed.

The results of multivariate regression with the step by step method shows that inattention to the positive issue, exaggerated generalization, musts and thinking of all, and no the highest explanation were 0.28, −0.22, −0.17, and 0.16, respectively. This means cognitive distortion with these amounts (0.28, −0.22, −0.17, and 0.16) can be predicted through religious orientation (Table 3).

**Table 3:** Multivariate regression results with the step by step method for predicting the amount of explanation of cognitive distortion through religious orientation

**Variable**	**T**	**β**	**SD** †	**Unstandardized coeffcients**	**Significant**
**Discounting the positive**	−4.66	−0.28	0.65	−3.05	0.00
**Overgeneralization**	−3.76	−0.22	0.51	−1.90	0.00
**Should statements**	−2.86	−0.17	0.70	−2.02	0.00
**All or nothing thinking**	−2.67	−0.16	0.52	−1.39	0.00

† Standard deviation

## Discussion

According to the results, the first hypothesis that expresses significant relationship between extrinsic religious orientation and cognitive orientation is confirmed. In other words, increased extrinsic religious orientation will increase the cognitive distortion in students. This result in-line with Albert Allis theory, who believed that religion, is related to irrational thought, religious individual have such dogmatic thoughts and behaviors that cause mental disorder and also these individuals have poor mental health religion, but it is different from intrinsic aspect of religion (15). The present research findings are confirmed (16-18). The extrinsic religion scale theoretical assess irrational belief, therefore should predict higher levels of unhealthy psychological reactivity. In fact, many studies have shown that extrinsic religion orientation is related to more anxiety (19) and irrational beliefs (20).

The second foundation that expresses there is a meaningful relationship between intrinsic religious orientation and cognitive distortion, is also confirmed. In the other word, whatever the rate of intrinsic religious orientation increase, the rate of cognitive distortion, decreases. In all kinds of cognitive errors, humans can read others mind, or they attempt to guess other’s emotions and beliefs. They have full trust in their guess, while they do not have absolute guess (21). This study is confirmed (22). Allport believes that intrinsic religious orientation is an exhaustive motivating commitment and not a tool to reach a personal goal. In other word, religious orientation equals as being religious (23). Therefore, the individual with intrinsic religious orientation cognitively more rational, have more mental health and are more satisfied with life (21).

The third foundation showed that there is no relationship between age and religious orientation. This result is confirmed (24-26). In explanation of these findings, we can mention to the Allport’s theory. Based on this theory, intrinsic religion is pervasive, organized and has been internalized. While the extrinsic religion is an extrinsic feature and is an instrument that is used for achieving to the personal needs such as security and position. Allport’s intrinsic religious orientation is a motivating exhaustive commitment which is the goal and not a tool to achieve personal purposes (27).

In general, the student with intrinsic religious orientation can overcome the cognitive distortions and decrease the amount of cognitive distortion, while the students also with extrinsic religious orientation because they use religion merely to access their goals, and have many cognitive errors and distortion. Finally, it could be concluded that intrinsic orientation to religion, as also found in the present study that influenced on individual cognition, cause reduces the use of cognitive distortions. Limitation of this study includes a self-reported tool (questionnaire) and the use of Muslim student sample. Hence, results are not generalizable to other communities. It is suggested that this research should be conducted on other individuals with different education levels and religions. Moreover, other variable can also be added to the research subject, that is, spiritual intelligence.

In relation to the practical dimension of results, also we could offer that, having multiple cognitive distortions could affect interpersonal relationships and various psychiatric disorders especially anxiety disorders. So by reinforcing individuals in the field of internalize religion we could make a step toward reducing these issues. More emphasis on parenting styles in building a platform for increasing inter-religious orientation could be one of the applications of this research. 

## Authors' contributions

LA conceived and designed the evaluation collected the clinical data, interpreted the clinical data, performed the statistical analysis, drafted the manuscript, revised it critically for important intellectual content, Read and approve the final manuscript; SHA collected the clinical data, interpreted the clinical data, performed the statistical analysis; AE Read and approve the final manuscript.

## Notes

**Declaration of interest:** None.

## References

[1] Martin D, Wrightsman S (1964). Religion and fears about death: a critical review of research. Religious Education.

[2] Yilmaz I (2002). The challenge of post-modern legality and Muslim legalpluralism in England. J Ethn Migr Stud.

[3] Bahrami H, Tashk A (2005). [The dimensions of the relationship between mental health and religious orientation and evaluation of the religious orientation scale]. J Psychol & Edu.

[4] Karami J, Roghanchi M, Attari Y, Beshlideh K, Shokri M (2006). [A study on simple relationships and religious orientation with the multiple dimensions of mental health in university students]. J Psychol Edu Sci.

[5] Eivazi MR (2002). [Earning on the cognitive approach]. Qabasat.

[6] Aghapour E, Mesri M (2011). [Relationship between religious orientation and mental health in family]. Quran and Medicine.

[7] Hosseini-Nasab SD, Hashemi T, Fotuhi-e Bonab S (2009). [Study of relationship between religious orientation and marital adjustment]. J Psychol.

[8] Warraich SA, Balchin C (2006). Recognizing the un-recognized: Inter-country cases and muslim marriages &amp; divorces in Britain [Online].

[9] Williams R (2008). Civil and religious law in England: a religious perspective. Ecc LJ.

[10] Zadhosh S, Neshatdost HT, Haghighat F, Rasolzadeh SK, Kalantari M (2011). [The effectiveness of group cognitive behavioral therapy with a religious orientation marital brrazaet ladies]. Stud Clin Psychol.

[11] Burns DD (1980). Feeling good: the new mood therapy.

[12] Bayani AA, Godarzi H, Godarzi A, Mohamad Kochaki A (2008). [Study on the relationship between religious orientation, anxiety and depression in medical students]. J Fundam Ment Health.

[13] Solati SK, Rabie M, Shariati M (2011). [The relationship between mental health and religious orientation]. Qom Univ Med Sci J.

[14] Mohamadzadeh RA, Babaei N, Yadolahi Z (2010). [Simple and multiple relationship between religious orientation and mental health].

[15] Chavoshi A, Talebian D, Tarkhorani H, Sedqi-Jalal H, Azarmi H, Fathi-Ashtiani A (2008). [The relationship between prayers and religious orientation with mental health]. J Behav Sci.

[16] Garavand H, Qanbari Hashimabadi BA, Kamkar zahirvand P, Jafari S (2011). [A comparative analysis of the relationship of religious orientation (intrinsic and extrinsic) with mental health and illogical beliefs]. Ravanshenasi Va Din.

[17] Sadiqi Arfa'ii F, Tamannaeifar MR, Abedinabadi A (2011). [The relationship between religious orientation, coping styles, and happiness of university students]. Ravanshenasi Va Din.

[18] Ehteshamzadeh P, Ahadi H, Enayati MS, Heidari A (2010). [Construct and validation of a scale for measuring interpersonal forgiveness]. Iran J Psychiatry Clin Psychol.

[19] Bahrami F, Ramezani Farani A (2005). [The role of intrinsic and extrinsic religious belief in geriatric mental health and depression rate]. Rehabilitation.

[20] Gorenstein EE, Newman JP (1980). Disinhibitory psychopathology: a new perspective and a model for research. Psychol Rev.

[21] Ali Akbari Dehkordi M, Oraki M, Barghi Irani Z (2010). [Relation between religious orientation with anxiety about death, and alienation in aged peoples (seniors) in Tehran]. Soc Psychol Res.

[22] Arefi M, Mohsenzadeh F (2011). [The relationship between religious orientation mental health, and gender]. J Women's Stu.

[23] Khdarhimi S, Jafari GR (1999). [The role of religion on mental health, psychotherapy and clinical psychology]. J Ment Hlth.

[24] Mansournezhad Z, Kajbaf MB, Kiani F, Pourseyed R (2012). [The relationship of religious orientation (intrinsic, extrinsic) and gender with death anxiety among students]. Res Cogn Behav Sci.

[25] Alizadeh Sahraee O, Khosravi Z, Besharat MA (2010). [The relation of irrational beliefs with positive and negative perfectionism among students in Nowshahr]. Q J Psychol Stud.

[26] Hosseinkhanzadeh AA, Hemati Alamdarloo G, Aghababaei H, Moradi A, Rezayi Dehnavi S (2011). [Prediction of self control capacity based on various religious orientations and its role in crime prevention]. Res Crim Law.

[27] Klerman GL, Weissman MM (2000). Interpersonal psychotherapy of depression.

